# Supplementary material for: Gene Expression in the Developing Seed of Wild and Domesticated Rice
Source: Int J Mol Sci. 2022 Nov 1;23(21):13351. doi: 10.3390/ijms232113351 (PMC9658725; doi:10.3390/ijms232113351)
Supplement: Supplementary file 1 [file ijms-23-13351-s001.zip › ijms-1982228-supplementary.pdf]

Supplementary Materials

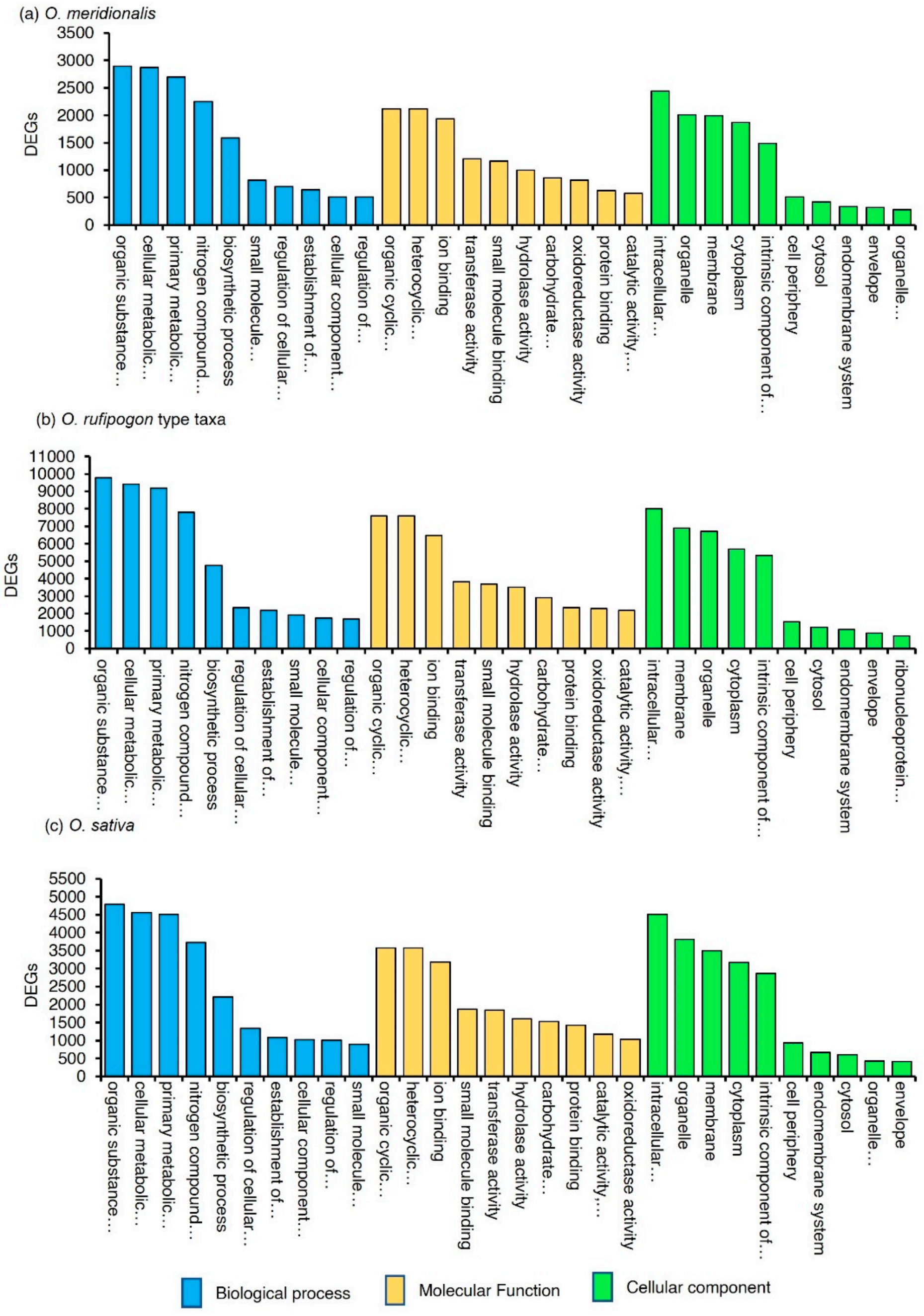

Figure S1 Top ten gene ontology (GO) terms represented by the highest number of differentially expressed genes (DEGs) for the three functional categories of gene ontology annotation (cellular component, molecular function, and biological process): (a) GO classification for the DEGs of *O. meridionalis*; (b) GO classification for the DEGs of *O. rufipogon* type taxa, and (c) GO classification for the DEGs of *O. sativa*

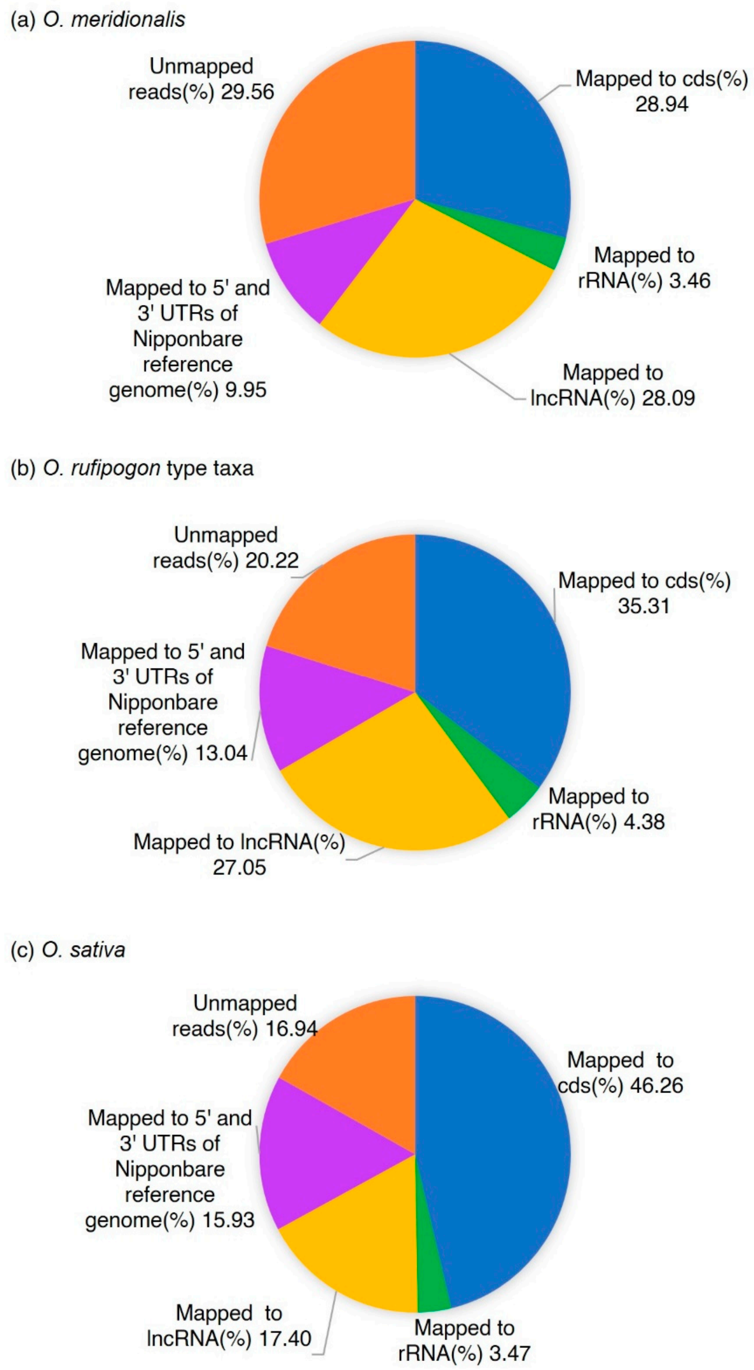

Figure S2 Mapping output of pooled RNA-seq reads from seed developing stages: (a) *O. meridionalis*, (b) *O. rufipogon* type taxa, and (c) *O. sativa*. Pooled RNA-Seq reads were mapped to coding sequences (CDS) of Os-Nipponbare-Reference-IRGSP-1.0 [31], ribosomal RNA (rRNA) database [51], long non-coding RNA (lncRNA) database [52], and the 3’ and 5’ UTRs (untranslated regions) of Os-Nipponbare-Reference-IRGSP-1.0 genome, respectively using CLC-GWB version 20 software

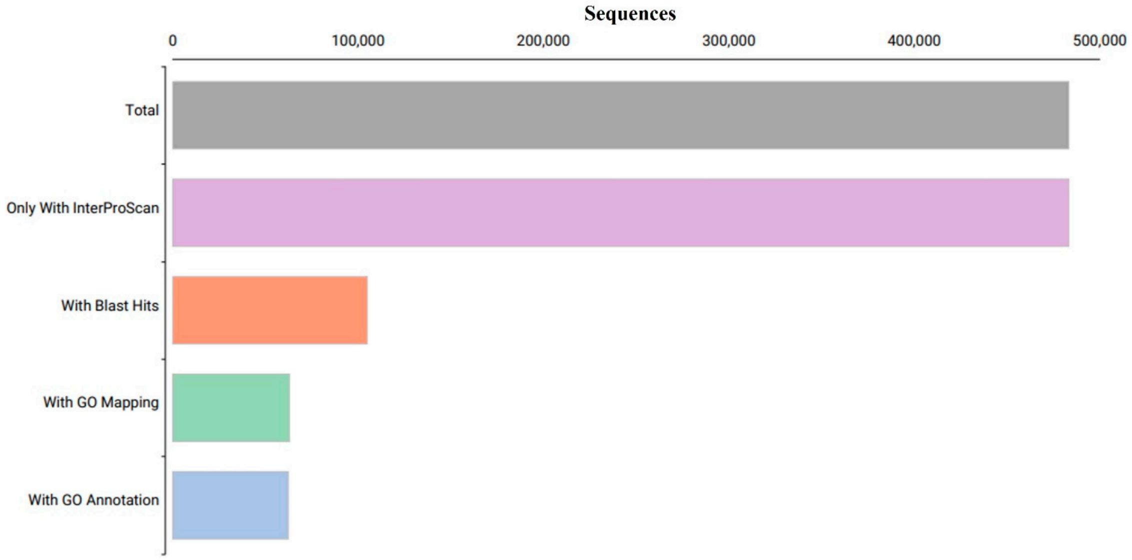

Figure S3 The output of BLAST (Basic Local Alignment Search Tool) analysis of 483,274 un-mapped sequences derived from *O. meridionalis*, *O. rufipogon* type taxa, and *O. sativa*

Table S1. Mapping output of 39 samples from *O. meridionalis*, *O. rufipogon type taxa*, and *O. sativa*. Reads were mapped to a new reference genome with 525,612 sequences consisting of CDS (coding sequences) of Os-Nipponbare-Reference-IRGSP-1.0 [31], long non-coding RNA (lncRNA), unmapped transcripts using CLC-GWB version 20 software. 39 samples from *O. meridionalis* (15 samples), *O. rufipogon* type taxa (15 samples), and *O. sativa* (9 samples). DPA denotes days post anthesis. R1, R2, R3, and R4 denote replicate 1, replicate 2, replicate 3 and replicate 4, respectively. .

Species	Sample ID	Total number of trimmed reads	Total number of mapped reads	Total number of unmapped reads	Mapped reads (%)	Unmapped reads (%)
O. meridionalis	5DPA-R1	57,646,086	45,870,708	11,775,378	79.57	20.43
	5DPA-R2	65,079,074	52,389,229	12,689,845	80.50	19.50
	5DPA-R3	80,270,325	67,087,191	13,183,134	83.58	16.42
	10DPA-R1	79,901,132	61,336,939	18,564,193	76.77	23.23
	10DPA-R2	82,935,992	67,673,536	15,262,456	81.60	18.40
	10DPA-R3	70,017,320	55,215,358	14,801,962	78.86	21.14
	15DPA-R1	75,220,637	52,415,055	22,805,582	69.68	30.32
	15DPA-R2	79,378,381	61,617,228	17,761,153	77.62	22.38
	15DPA-R3	78,290,265	65,191,196	13,099,069	83.27	16.73
	20DPA-R1	75,656,172	55,648,866	20,007,306	73.55	26.45
	20DPA-R2	79,768,886	62,664,619	17,104,267	78.56	21.44
	20DPA-R3	80,173,110	64,270,527	15,902,583	80.16	19.84
	25DPA-R1	80,784,269	34,216,083	46,568,186	42.35	57.65
	25DPA-R2	67,431,735	51,341,346	16,090,389	76.14	23.86
	25DPA-R3	87,398,528	68,554,210	18,844,318	78.44	21.56
O. rufipogon type taxa	5DPA-R1	80,695,578	61,841,641	18,853,937	76.64	23.36
	5DPA-R2	58,851,833	46,419,022	12,432,811	78.87	21.13
	5DPA-R3	68,095,707	54,117,338	13,978,369	79.47	20.53
	5DPA-R4	61,703,499	49,706,198	11,997,301	80.56	19.44
	10DPA-R1	80,643,321	65,960,827	14,682,494	81.79	18.21
	10DPA-R2	75,866,203	57,081,803	18784400	75.24	24.76
	10DPA-R3	80,399,913	61,426,407	18,973,506	76.40	23.60
	10DPA-R4	75,350,385	57,496,733	17,853,652	76.31	23.69
	15DPA-R1	84,533,570	62,736,736	21,796,834	74.22	25.78
	15DPA-R2	68,998,248	48,428,315	20,569,933	70.19	29.81
	15DPA-R3	82,253,874	62,267,809	19,986,065	75.70	24.30
	15DPA-R4	84,819,861	60,993,591	23,826,270	71.91	28.09

	25DPA-R1	73,408,716	54,312,244	19,096,472	73.99	26.01
	25DPA-R2	99,204,328	72,530,370	26,673,958	73.11	26.89
	25DPA-R3	99,355,437	69,336,746	30,018,691	69.79	30.21
O. sativa	5DPA-R1	75,105,371	59,698,634	15,406,737	79.49	20.51
	5DPA-R2	79,552,229	61,966,050	17,586,179	77.89	22.11
	5DPA-R3	83,679,467	67,218,116	16,461,351	80.33	19.67
	15DPA-R1	83,138,926	61,892,357	21,246,569	74.44	25.56
	15DPA-R2	61,789,583	44,712,234	17,077,349	72.36	27.64
	15DPA-R3	68,306,256	54,3338,23	13,972,433	79.54	20.46
	25DPA-R1	76,835,927	58,753,160	18,082,767	76.47	23.53
	25DPA-R2	54,964,733	40,919,710	14,045,023	74.45	25.55
	25DPA-R3	76,764,893	58,389,159	18,375,734	76.06	23.94

Table S2. Normalised expression of genes related to domestication traits (*qSH1*: *Shattering (QTL)-1*; *SH4: shattering 4*; *OsSh1*: *shattering 1; GS5*: *grain size 5*; *GS3*: *grain size 3*; *Bh4*: *black hull 4*; *Rc: brown pericarp and seed coat*), starch and sucrose metabolism (*AGPS1: ADP-glucose pyrophosphorylase small subunit 1*; *AGPS2*: *ADP glucose pyrophosphorylase small subunit 2*; *GBSSI*: *Granule-bound starch synthase I* ; *SSIIa*: *Soluble starch synthase IIa*; *BEIIb: Starch branching enzyme IIb*; *ISA1*: *Isoamylase 1*; *PHOL*: *Starch phosphorylase*; *SuSy1*: *Sucrose synthase 1*), and seed storage proteins (; *GluA-1*: *Glutelin type A-1*; *GluB-1a*: *Glutelin type B-1a*; *GluC-1*: *Glutelin type C-1*; *GluD-1*: *Glutelin type D-1*). Data are presented as average of the reads per kilobase of transcript per million reads mapped (RPKM) values ± standard deviation of three biological replicates for *O. meridionalis* and *O. sativa* and four biological replicates for *O. rufipogon* type taxa (except for 25 DPA with three replicates) at seed development stages. DPA denotes days post anthesis.

Genes	*O. meridionalis*					*O. rufipogon* type taxa				*O. sativa*		
	5 DPA	10 DPA	15 DPA	20 DPA	25 DPA	5 DPA	10 DPA	15 DPA	25 DPA	5 DPA	15 DPA	25 DPA
qSH1	6.55	3.08	2.93	1.47	2.04	9.11	4.61	2.98	1.88	6.91	5.36	8.25
	(±0.37)	(±1.17)	(±0.54)	(±0.33)	(±0.83)	(±0.28)	(±0.73)	(±1.06)	(±0.57)	(±1.38)	(±1.95)	(±2.44)
SH4	0.55	0.30	0.08	0.01	0.06	0.72	0.46	0.43	0.08	0.26	0.49	0.16
	(±0.08)	(±0.24)	(±0.05)	(±0.02)	(±0.06)	(±0.20)	(±0.35)	(±0.32)	(±0.08)	(±0.15)	(±0.61)	(±0.07)
OsSh1	25.12	13.67	12.51	4.43	2.96	21.16	11.80	9.04	2.31	25.71	16.51	5.76
	(±2.74)	(±3.62)	(±3.40)	(±2.18)	(±1.51)	(±1.87)	(±2.15)	(±2.80)	(±0.87)	(±5.49)	(±9.85)	(±2.90)
GS5	1.55	0.59	0.50	0.02	0.15	2.47	0.81	0.38	0.13	1.64	1.11	0.40
	(±0.37)	(±0.15)	(±0.03)	(±0.01)	(±0.09)	(±0.86)	(±0.36)	(±0.18)	(±0.17)	(±0.23)	(±0.98)	(±0.41)
GS3	0.18	0.16	0.18	0.09	0.32	0.23	0.30	0.49	0.14	0.44	1.05	0.93
	(±0.23)	(±0.17)	(±0.32)	(±0.06)	(±0.15)	(±0.14)	(±0.38)	(±0.21)	(±0.07)	(±0.29)	(±0.98)	(±0.50)
Bh4	0.07	10.40	43.19	33.16	22.65	0.19	7.33	27.23	10.84	0.01	8.75	6.53
	(±0.11)	(±2.54)	(±24.72)	(±9.86)	(±9.98)	(±0.31)	(±2.42)	(±11.97)	(±7.27)	(±0.01)	(±2.89)	(±3.64)
Rc	14.17	7.30	4.52	0.62	0.31	33.28	18.52	8.92	0.79	2.36	0.77	0.27
	(±2.97)	(±3.71)	(±1.17)	(±0.79)	(±0.38)	(±1.97)	(±5.14)	(±2.79)	(±1.21)	(±0.28)	(±0.19)	(±0.24)

AGPS1	66.13	23.87	18.26	5.74	8.89	77.84	47.23	16.75	5.24	150.39	46.76	55.07
	(±8.51)	(±5.34)	(±3.32)	(±2.21)	(±1.95)	(±10.12)	(±12.33)	(±5.78)	(±2.67)	(±15.98)	(±11.72)	(±3.41)
AGPS2	837.70	460.29	338.84	123.37	127.34	1,239.8	1,077.5	599.92	261.55	935.19	859.51	444.92
	(±150.91)	(±94.79)	(±126.97)	(±66.25)	(±48.57)	(±102.31)	(±242.13)	(±140.04)	(±106.57)	(±100.61)	(±54.74)	(±119.95)
GBSSI	3,033.4	3,798.5	3,549.9	2,023.8	2,111.5	2,457.7	3,945.4	3,226.9	2,094.7	675.56	398.82	156.55
	(±1018.9)	(±1245.9)	(±2120.6)	(±577.63)	(±117.49)	(±417.08)	(±1023.5)	(±724.73)	(±639.26)	(±135.21)	(±92.0)	(±48.11)
SSIIa	122.45	66.74	49.42	12.42	14.99	178.89	126.81	50.51	12.36	133.44	75.10	26.58
	(±51.67)	(±13.66)	(±18.83)	(±2.79)	(±1.95)	(±24.13)	(±51.93)	(±17.0)	(±9.56)	(±11.41)	(±7.90)	(±11.30)
BEIIb	765.17	480.56	318.55	100.91	122.74	1,265.6	841.84	379.45	107.10	802.13	350.08	108.85
	(±132.74)	(±77.03)	(±49.73)	(±48.70)	(±16.54)	(±149.55)	(±322.23)	(±118.22)	(±61.89)	(±92.33)	(±74.11)	(±43.21)
ISA1	159.01	96.69	65.57	12.85	16.21	193.81	129.59	54.55	7.93	181.86	51.67	8.59
	(±35.96)	(±21.47)	(±13.24)	(±10.19)	(±3.36)	(±25.34)	(±39.95)	(±16.99)	(±8.16)	(±29.56)	(±23.82)	(±2.29)
PHOL	384.93	190.46	120.03	18.42	22.39	918.92	600.68	221.10	35.50	513.78	223.64	53.84
	(±84.33)	(±70.05)	(±59.01)	(±9.80)	(±2.77)	(±115.77)	(±235.41)	(±67.08)	(±35.16)	(±81.90)	(±39.15)	(±23.94)
SuSy1	88.12	40.28	28.28	2.69	6.54	70.46	39.02	14.49	3.47	80.30	16.97	5.66
	(±13.14)	(±5.45)	(±8.75)	(±0.23)	(±2.51)	(±2.69)	(±16.77)	(±5.76)	(±3.34)	(±18.78)	(±10.73)	(±1.32)
GluA-1	18,068.1	23,123.8	12,557.5	4,634.8	4,298.3	9,341.2	13,315.0	9,756.50	3,059.6	16,662.9	14,046.8	5,039.3
	(±6929.7)	(±11672)	(±8296.4)	(±2957.7)	(±2075.3)	(±2279.7)	(±3697.8)	(±2519.9)	(±893.11)	(±1678.8)	(±3351.8)	(±2240.5)
GLuB-1a	12,196.9	10,006.8	8,062.0	1,905.16	2,006.4	9,763.9	10,307.9	6,712.5	2,979.5	7,045.9	5,421.8	2,018.7
	(±4813.6)	(±4979.8)	(±6904.5)	(±771.20)	(±583.40)	(±1777.03)	(±1261.6)	(±767.15)	(±628.27)	(±1232.8)	(±1793.1)	(±1494.9)
GluC-1	5,140.1	4,024.1	1,280.8	660.3	824.8	4,106.3	4,166.5	2,398.9	642.6	4,453.1	3,504.4	1,978.5
	(±647.16)	(±1009.18)	(±769.80)	(±367.36)	(±230.79)	(±509.93)	(±1004.5)	(±615.12)	(±242.22)	(±884.41)	(±204.33)	(±431.86)
GluD-1	523.14	435.33	227.61	117.44	102.60	1,359.52	1,222.01	786.65	311.74	1,000.8	1,540.8	681.27
	(±800.3)	(±656.21)	(±346.21)	(±181.92)	(±166.01)	(±241.57)	(±341.92)	(±225.78)	(±81.05)	(±61.29)	(±224.22)	(±295.68)

Table S3 Upregulation and down-regulation of DEGs associated with domestication traits in *O. meridionalis*, *O. rufipogon* type taxa, and *O. sativa*: *shattering 1* (*OsSh1*); *grain size 5* (*GS5*); *black hull 4* (*Bh4*); *brown pericarp and seed coat* (*Rc*). The positive fold change value denotes up-regulation, and the negative fold change value represents down-regulation. DEGs are significant at a false discovery rate (FDR) p-value of ≤ 0.01. DPA denotes days post anthesis. DPA on the right side of a comparison group represents a control group.

	O. meridionalis		*O. rufipogon* type taxa		*O. sativa*	
Gene	Comparison group (fold change value)	Expression stages	Comparison group (fold change value)	Expression stages	Comparison group (fold change value)	Expression stages
shattering 1 (*OsSh1*)	25DPAvs5DPA (-5.63)	5DPA	25DPAvs5DPA (-3.56)	5DPA	25DPAvs5DPA (-5.11)	5DPA
grain size 5 (*GS5*)	-	-	15DPA vs 5DPA (-6.34)<br>25DPA vs 5DPA (-8.57)	5DPA	25DPAvs5DPA (-5.22)	5DPA
black hull 4 (*Bh4*)	10DPAvs5DPA (4,752.97)<br>15DPAvs5DPA (22456.34)<br>20DPAvs5DPA (28,612.09)<br>25DPAvs5DPA (11466.12)	10DPA<br>15DPA<br>20DPA<br>25DPA	15DPAvs5DPA (183.57)<br>25DPAvs5DPA (145.81)<br>10DPAvs5DPA (55.11)	10DPA<br>15DPA<br>25DPA	15DPAvs5DPA (784.18)<br>25DPAvs5DPA (551.84)	15DPA-25DPA
brown pericarp and seed coat (*Rc*)	25DPAvs5DPA (-25.67)<br>20DPAvs5DPA (-9.92)<br>25DPAvs10DPA (-15.67)	5DPA<br>10DPA	15DPAvs5DPA (-3.07)<br>25DPAvs5DPA (-20.41)<br>25DPAvs10DPA (-16.59)	5DPA<br>10DPA	25DPAvs5DPA (-10.2)	5DPA

Table S4 Up-regulation and down-regulation of differentially expressed genes (DEGs) for starch and sucrose metabolism in *O. meridionalis*, *O. rufipogon* type taxa, and *O. sativa*: *ADP-glucose pyrophosphorylase small subunit 1*(*AGPS1*); *ADP glucose pyrophosphorylase small subunit 2* (*AGPS2*); *Granule-bound starch synthase I* (*GBSSI*); *Soluble starch synthase IIa* (*SSIIa*); *Starch branching enzyme IIb* (*BEIIb*); *Isoamylase 1* (*ISA1*); *Starch phosphorylase* (*PHOL*); *Sucrose synthase 1* (*SuSy1*). The positive fold change value denotes up-regulation, and the negative fold change value represents down-regulation. DEGs are significant at a false discovery rate (FDR) p-value of ≤ 0.01. DPA denotes days post anthesis. DPA on the right side of a comparison group represents a control group.

**Species**	***O. meridionalis***		***O. rufipogon* type taxa**		***O. sativa***	
Gene	Comparison group (Fold change value)	Expression stages	Comparison group (Fold change value)	Expres-sion stages	Comparison group (Fold change value)	Expres-sion stages
ADP-glucose pyrophosphorylase small subunit 1(AGPS1)	20DPAvs5DPA (-4.74) 25DPAvs5DPA (-4.99)	5DPA	15DPAvs5DPA (-3.98) 25DPAvs5DPA (-6.06) 25DPAvs10DPA (-5.31)	5DPA 10DPA	25DPAvs5DPA (-2.99)	5DPA
ADP glucose pyrophosphorylase small subunit 2 (AGPS2)	25DPAvs5DPA (-4.18)	5DPA	-		25DPAvs5DPA (-2.26)	5DPA
Granule-bound starch synthase I (GBSSI)	-	-	-	-	25DPAvs5DPA (-4.56)	5DPA
Soluble starch synthase IIa (SSIIa)	25DPAvs5DPA (-5.51) 20DPAvs5DPA (-4.18)	5DPA	15DPAvs5DPA (-2.94) 25DPAvs5DPA (-6.21) 25DPAvs10DPA (-6.35)	5DPA 10DPA	25DPAvs5DPA (-5.40)	5DPA
Starch branching enzyme IIb (BEIIb)	25DPAvs5DPA (-4.03)	5DPA	15DPAvs5DPA (-2.81) 25DPAvs5DPA (-4.85) 25DPAvs10DPA (-4.66)	5DPA 10DPA	25DPAvs5DPA (-7.85)	5DPA
Isoamylase 1 (ISA1)	25DPAvs5DPA (-6.29)	5DPA	15DPAvs5DPA (-2.97) 25DPAvs5DPA	5DPA 10DPA	25DPAvs5DPA (-23.02)	5 DPA 15DPA

	20DPAvs5DPA<br>(-5.38)		(-10.86)<br>25DPAvs10DPA<br>(-10.57)		25DPAvs15DPA<br>(-6.05)	
Starch phosphorylase (PHOL)	20DPAvs5DPA<br>(-8.99)<br>25DPAvs5DPA<br>(-11.43)<br>25DPAvs10DPA<br>(-6.47)	5DPA<br>10DPA	15DPAvs5DPA<br>(-3.53)<br>25DPAvs5DPA<br>(-11.28)<br>25DPAvs10DPA<br>(-10.61)	5DPA<br>10DPA	25DPAvs5DPA<br>(-10.24)	5DPA
Sucrose synthase 1 (SuSy1)	25DPAvs5DPA<br>(-3.10)<br>20DPAvs5DPA<br>(-13.31) 20DPAvs10DPA<br>(-7.77) 20DPAvs15DPA<br>(-5.91)	5DPA<br>10DPA<br>15DPA	15DPAvs5DPA<br>(-4.01) 25DPAvs5DPA<br>(-8.71) 25DPAvs10DPA<br>(-7.02)	5DPA<br>10DPA	25DPAvs5DPA<br>(-15. 53)	5 DPA

Table S5. Discovery of novel transcripts from BLAST (Basic Local Alignment Search Tool) analysis of unmapped transcripts from three species (*O. meridionalis*, *O. rufipogon* type taxa, and *O. sativa*). A total of 38 novel transcripts associated with hull colour (*black hull 4*), starch and sucrose metabolism (*Granule-bound starch synthase I*; *Granule-bound starch synthase II*; *Soluble starch synthase I*; *Soluble starch synthase IIIa*; *Soluble starch synthase IVa*; *Starch branching enzyme I*; *Starch branching enzyme IIa*; *Starch branching enzyme IIb*; *Isoamylase 1*; *Pullulanase*; *Starch phosphorylase*; *Sucrose synthase 2*; *Sucrose synthase 3*; *Sucrose synthase 6*), and seed storage proteins (*Glutelin*; *Glutelin type-A 1*; *Glutelin type-A 2*; *Glutelin type-B 5*). Complete: with both start and stop codon; 3'partial: with missing stop codon and presumably part of the C-terminus; 5'partial: with missing start codon and presumably part of the N-terminus; internal: with both start and stop codons missing.

Genes	Number of novel transcripts	Unmapped transcript ID	Length (bp)	Coding potential
black hull 4	1	WRiceTA25_R2_contig_6402	823	5'partial
Granule-bound starch synthase I	2	WRiceTA25_R2_contig_131873	286	internal
		WRiceTA25_R2_contig_281548	160	internal
Granule-bound starch synthase II	1	WRiceTA25_R2_contig_274153	192	internal
Soluble starch synthase I	4	RiceNIP25_R2_contig_103765	164	internal
		WRiceTA25_R2_contig_101243	164	internal
		WRiceTA25_R2_contig_23105	2,985	complete
		RiceNIP25_R2_contig_203515	181	internal
Soluble starch synthase IIIa	3	RiceNIP25_R2_contig_202233	222	internal
		WRiceTA25_R2_contig_269931	279	5' partial
		WRiceTA25_R2_contig_299890	200	internal
Soluble starch synthase IVa	3	RiceNIP25_R2_contig_138892	271	internal
		WRiceTA25_R2_contig_15451	306	internal
		WRiceTA25_R2_contig_89681	212	internal
Starch branching enzyme I	3	WRiceTA25_R2_contig_248515	289	internal
		RiceNIP25_R2_contig_189222	234	internal
		RiceNIP25_R2_contig_181958	361	internal
Starch branching enzyme IIa	3	WRiceTA25_R2_contig_24197	550	complete
		RiceNIP25_R2_contig_15492	390	internal
		WRiceTA25_R2_contig_30137	216	internal
Starch branching enzyme IIb	1	WRiceTA25_R2_contig_108471	186	internal
Isoamylase I	1	WRiceTA25_R2_contig_239384	477	internal
Pullulanase	1	WRiceTA25_R2_contig_252	2,051	complete
Starch phosphorylase	2	RiceNIP25_R2_contig_176856	196	internal
		WRiceTA25_R2_contig_131708	390	5'partial
Sucrose synthase 2	2	RiceNIP25_R2_contig_218706	234	internal
		WRiceTA25_R2_contig_154522	209	internal
Sucrose synthase 3	1	RiceNIP25_R2_contig_209729	184	internal
Sucrose synthase 6	1	RiceNIP25_R2_contig_211160	163	internal
Glutelin	3	RiceNIP25_R2_contig_53290	172	internal
		RiceNIP25_R2_contig_124528	229	3'partial
		RiceNIP25_R2_contig_179620	320	5'partial
Glutelin type-A 1	2	WRiceTA25_R2_contig_52031	321	internal
		WRiceTA25_R2_contig_239042	172	internal
Glutelin type-A 2	1	RiceNIP25_R2_contig_6983	343	complete
Glutelin type-B 5	3	RiceNIP25_R2_contig_193984	233	3'partial
		WRiceTA25_R2_contig_125425	264	5'partial
		WRiceTA25_R2_contig_145934	315	5'partial

Table S6 Mapping output of 39 samples from *O. meridionalis*, *O. rufipogon type taxa*, and *O. sativa*. Reads were mapped to CDS (coding sequences) of Os-Nipponbare-Reference-IRGSP-1.0 [31] using CLC-GWB version 20 software. R1, R2, R3, and R4 denote replicate 1, replicate 2, replicate 3, and replicate 4, respectively. DPA denotes days post anthesis.

Species	Samples ID	Total number of trimmed reads	Total number of mapped reads to CDS	Total number of unmapped reads to CDS	Mapped reads (%)	Unmapped reads (%)
O. meridionalis	5DPA-R1	57,646,086	26,966,418	30,679,668	46.78	53.22
	5DPA-R2	65,079074	26,287,187	38,791,887	40.39	59.61
	5DPA-R3	80,270,325	46,771,464	33,498,861	58.27	41.73
	10DPA-R1	79,901,132	27,305,338	52,595,794	34.17	65.83
	10DPA-R2	82,935,992	24,805,500	58,130,492	29.91	70.09
	10DPA-R3	70,017,320	37,553,039	32,464,281	53.63	46.37
	15DPA-R1	75,22,0637	14,380,188	60,840,449	19.12	80.88
	15DPA-R2	79,378,381	17,789,979	61,588,402	22.41	77.59
	15DPA-R3	78,290,265	34,075,435	44,214,830	43.52	56.48
	20DPA-R1	75,656,172	11,347,565	64,308,607	15.00	85.00
	20DPA-R2	79,768,886	80,098,93	71,758,993	10.04	89.96
	20DPA-R3	80,173,110	14,492,077	65,681,033	18.08	81.92
	25DPA-R1	80,784,269	11,703,252	69,081,017	14.49	85.51
	25DPA-R2	67,431,735	10,713,392	56,718,343	15.89	84.11
	25DPA-R3	87,398,528	17,737,368	69,661,160	20.29	79.71
O. rufipogon type taxa	5DPA-R1	80,695,578	42,067,561	38,628,017	52.13	47.87
	5DPA-R2	58,851,833	31,298,095	27,553,738	53.18	46.82
	5DPA-R3	68,095,707	38,665,976	29,429,731	56.78	43.22
	5DPA-R4	61,703,499	35,704,593	25,998,906	57.86	42.14
	10DPA-R1	80,643,321	44,439,014	36,204,307	55.11	44.89
	10DPA-R2	75,866,203	27,351,167	48,515,036	36.05	63.95
	10DPA-R3	80,399,913	29,029,813	51,370,100	36.11	63.89
	10DPA-R4	75,350,385	26,357,386	48,992,999	34.98	65.02
	15DPA-R1	84,533,570	26,059,304	58,474,266	30.83	69.17
	15DPA-R2	68,998,248	14,416,695	54,581,553	20.89	79.11
	15DPA-R3	82,253,874	27,301,789	54,952,085	33.19	66.81
	15DPA-R4	84,819,861	26,164,480	58,655,381	30.85	69.15
	25DPA-R1	73408716	17245618	56163098	23.49	76.51
	25DPA-R2	99,204,328	13,604,698	85,599,630	13.71	86.29
	25DPA-R3	99,355,437	14,937,955	84,417,482	15.03	84.97
O. sativa	5DPA-R1	75,105,371	41,849,463	33,255,908	55.72	44.28
	5DPA-R2	79,552,229	33,585,272	45,966,957	42.22	57.78
	5DPA-R3	83,679,467	45,879,842	37,799,625	54.83	45.17
	15DPA-R1	83,138,926	33,182,103	49,956,823	39.91	60.09
	15DPA-R2	61,789,583	19,196,897	42,592,686	31.07	68.93
	15DPA-R3	68,306,256	37,458,729	30,847,527	54.84	45.16
	25DPA-R1	76,835,927	38,269,316	38,566,611	49.81	50.19
	25DPA-R2	54,964,733	24,434,205	30,530,528	44.45	55.55
	25DPA-R3	76,764,893	31,516,314	45,248,579	41.06	58.94

Table S7 List of 19 long non-coding RNA (lncRNA). These lncRNAs were obtained from plant long non-coding RNA (lncRNA) database [51]

Sequences	Consensus length	Total read count	Average coverage	Reference sequence	Reference length	Coverage†
TCONS_00055850 mapping	467	1409	280.7784946	TCONS_00055850	465	100
lincRNA430_PMID_24635777 mapping	4286	94414	2182.538838	lincRNA430_PMID_24635777	4287	99.98
TCONS_00018605 mapping	254	834	291.8935361	TCONS_00018605	263	96.58
NONATHT001142 mapping	1499	5960	353.5490699	NONATHT001142	1559	96.15
NONATHT001134 mapping	2196	4243	173.3494392	NONATHT001134	2318	94.74
lincRNA11_PMID_24635777 mapping	11470	61021	462.3476697	lincRNA11_PMID_24635777	13067	87.78
lincRNA297_PMID_24635777 mapping	320	36356565	9214691.597	lincRNA297_PMID_24635777	367	87.19
NONATHT001141 mapping	761	50453	5644.563636	NONATHT001141	880	86.48
NONATHT001128 mapping	217	1863	638.4140625	NONATHT001128	256	84.77
NONATHT001130 mapping	749	7242	785.1742081	NONATHT001130	884	84.73
XLOC_000829 mapping	464	23	4	XLOC_000829	562	82.56
NONATHT001127 mapping	1865	12510	533.2854633	NONATHT001127	2277	81.91
XLOC_004531 mapping	191	14	4.817073171	XLOC_004531	246	77.64
XLOC_001235 mapping	160	5	1.769230769	XLOC_001235	208	76.92
NONATHT001131 mapping	4566	15193	243.6844848	NONATHT001131	5949	76.75
NONATHT001136 mapping	163	1152	408.7351598	NONATHT001136	219	74.43
NONATHT001126 mapping	4452	44619	702.7148276	NONATHT001126	6063	73.43
lincRNA688_PMID_24635777 mapping	3779	8956	169.5256311	lincRNA688_PMID_24635777	5150	73.38
NONATHT001117 mapping	277	284	67.41902314	NONATHT001117	389	71.21

† coverage was calculated as (consensus length/reference length)*100.

Table S8 Different comparison groups and control groups for differential expression analysis in two wild (*O. meridionalis* and *O. rufipogon* type taxa) and one cultivated rice (*O. sativa* ssp. *japonica* var. Nipponbare). 10 comparison groups were made from five seed developing stages (5 DPA, 10 DPA, 15 DPA, 20 DPA, and 25DPA) in *O. meridionalis*. Six comparison groups were derived from four seed developing stages (5 DPA, 10 DPA, 15 DPA, and 25 DPA) in *O. rufipogon* type taxa. Three comparison groups were made from three seed developing stages (5 DPA, 10 DPA, and 25 DPA) in *O. sativa*.

Species	Comparison groups	Control group
O. meridionalis	10 DPA vs 5 DPA	5 DPA
	15 DPA vs 5 DPA	5 DPA
	20 DPA vs 5 DPA	5 DPA
	25 DPA vs 5 DPA	5 DPA
	15 DPA vs 10 DPA	10 DPA
	20 DPA vs 10DPA	10 DPA
	25 DPA vs 10 DPA	10 DPA
	20 DPA vs 15 DPA	15 DPA
	25 DPA vs 15 DPA	15 DPA
	25 DPA vs 20 DPA	20 DPA
O. rufipogon type taxa	10 DPA vs 5 DPA	5 DPA
	15 DPA vs 5 DPA	5 DPA
	25 DPA vs 5 DPA	5 DPA
	15 DPA vs 10 DPA	10 DPA
	25 DPA vs 10 DPA	10 DPA
	25 DPA vs 15 DPA	15 DPA
O. sativa	15 DPA vs 5 DPA	5 DPA
	25 DPA vs 5 DPA	5 DPA
	25 DPA vs 15DPA	15 DPA
